# Atrial Functional Tricuspid Regurgitation: A Comprehensive Review of Pathophysiology, Diagnosis, and Management Strategies

**DOI:** 10.31083/j.rcm2512435

**Published:** 2024-12-11

**Authors:** Moiud Mohyeldin, Ahmed Abdelghafar, Sai Allu, Shitij Shrivastava, Ahmed Mustafa, Feras O. Mohamed, Sarah J. Norman

**Affiliations:** ^1^Department of Medicine, BronxCare Health System, Bronx, NY 10457, USA; ^2^Department of Medicine, University of Medical Sciences and Technology (UMST), 12810 Khartoum, Sudan; ^3^Department of Medicine, Salaam Clinic, Cleveland, OH 44106, USA; ^4^Department of Radiology, Texas Medical Center Memorial Hermann Hospital, Houston, TX 77030, USA; ^5^American University of the Caribbean School of Medicine, Cupecoy, Sint Maarten

**Keywords:** atrial functional tricuspid regurgitation, atrial fibrillation, right atrial remodeling, tricuspid annular dilatation, leaflet tethering, right ventricular function, surgical tricuspid valve repair, transcatheter tricuspid valve interventions, tricuspid valve replacement

## Abstract

Atrial fibrillation (AF), the most prevalent sustained cardiac arrhythmia, is intricately linked with atrial functional tricuspid regurgitation (AFTR), a condition distinguished from ventricular functional tricuspid regurgitation by its unique pathophysiological mechanisms and clinical implications. This review article delves into the multifaceted aspects of AFTR, exploring its epidemiology, pathophysiology, diagnostic evaluation, and management strategies. Further, we elucidate the mechanisms underlying AFTR, including tricuspid annular dilatation, right atrial enlargement, and dysfunction, which collectively contribute to the development of tricuspid regurgitation in the absence of significant pulmonary hypertension or left-sided heart disease. The section on diagnostic evaluation highlights the pivotal role of echocardiography, supplemented by cardiac magnetic resonance (CMR) imaging and computed tomography (CT), in assessing disease severity and guiding treatment decisions. Management strategies for AFTR are explored, ranging from medical therapy and rhythm control to surgical and percutaneous interventions, underscoring the importance of a tailored, multidisciplinary approach. Furthermore, the article identifies gaps in current knowledge and proposes future research directions to enhance our understanding and management of AFTR. By providing a comprehensive overview of AFTR, this review aims to raise awareness among healthcare professionals and stimulate further research to improve patient care and outcomes in this increasingly recognized condition.

## 1. Introduction

Atrial fibrillation (AF) is the most common sustained cardiac arrhythmia, affecting millions of people worldwide [1]. AF is associated with various cardiovascular complications, including stroke, heart failure, and valvular heart disease [2]. Recently, there has been growing recognition of the link between AF and isolated tricuspid regurgitation (TR), particularly atrial functional TR (AFTR) [3, 4].

AFTR differs from ventricular functional TR (VFTR), with different pathophysiological mechanisms and clinical implications [3, 5]. Secondary (or functional) TR results from the deformation of the tricuspid valve complex with morphologically normal leaflets. Moreover, secondary TR is mainly associated with right ventricular dilatation and/or dysfunction, annular dilatation, and/or leaflet tethering. These issues are usually secondary to left-sided valvular heart disease (especially affecting the mitral valve), atrial fibrillation, or pulmonary hypertension [6]. AFTR is characterized by TR secondary to right atrial enlargement and atrial cardiopathy, without significant pulmonary hypertension or left-sided heart disease [3, 7]. Despite its increasing prevalence and prognostic significance, AFTR remains an underappreciated and understudied condition [8, 9].

The prevalence of AF is increasing due to the aging population and improved life expectancy with chronic diseases [10]. In AFTR, TR is a surrogate marker of atrial cardiopathy, which precedes AF [11]. The natural history of TR and right heart chamber remodeling in patients with AF has been poorly assessed; however, restoring sinus rhythm appears beneficial for reducing TR severity and promoting reverse remodeling [12].

This review article aims to provide a comprehensive overview of AFTR, focusing on its pathophysiology, diagnostic evaluation, and management strategies. We discuss its epidemiology, association with AF, and impact on outcomes, highlighting key echocardiographic findings. We also address management in specific populations and summarize current treatment options, identifying gaps in understanding and proposing future research directions to enhance patient care.

## 2. Pathophysiology of AFTR

**Fig. 1.**
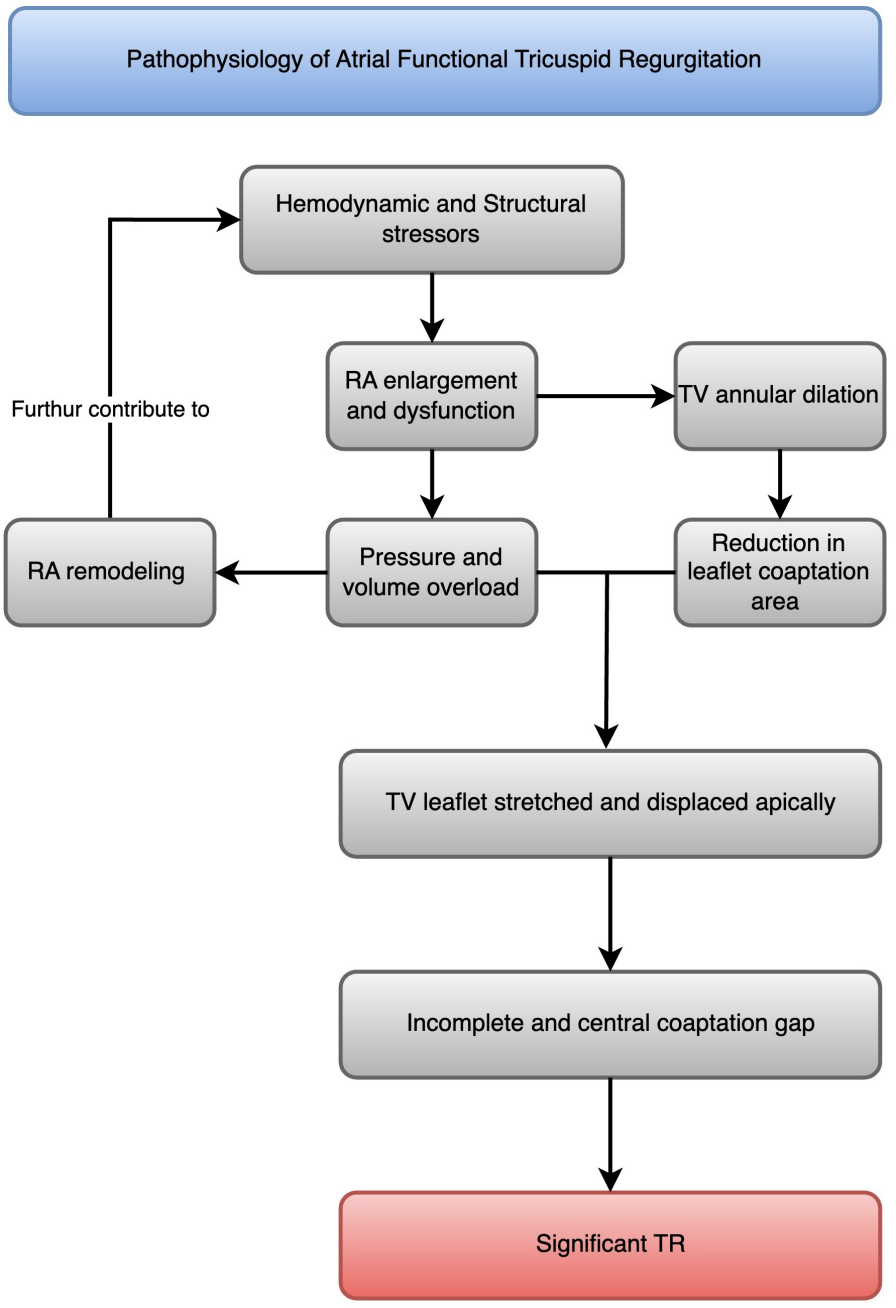
**Pathophysiology of atrial functional tricuspid regurgitation**. A flowchart that illustrates the progression from atrial fibrillation to right atrial enlargement and dysfunction, which leads to tricuspid annular dilatation, TV leaflet tethering, malcoaptation, and atrial functional tricuspid regurgitation (AFTR). RA, right atrial; TR, tricuspid regurgitation; TV, tricuspid valve.

AF is a common arrhythmia that can lead to various cardiovascular complications, including AFTR [3]. Indeed, AFTR is a distinct entity from VFTR (Fig. 1), with different pathophysiological mechanisms and clinical implications [3].

### 2.1 Mechanisms of TR in Patients with AF

#### 2.1.1 Right Atrial Enlargement and Dysfunction

In patients with AF, the right atrium undergoes progressive enlargement and dysfunction due to irregular and rapid electrical activity [13]. The structural changes in atrial myocytes caused by AF include (1) an increase in cell size, (2) accumulation of glycogen around the cell nucleus, (3) loss of sarcomeres in the center of the cell, (4) changes in connexin expression, (5) alterations in mitochondrial shape, (6) fragmentation of the sarcoplasmic reticulum, (7) even spread of nuclear chromatin, and (8) changes in the quantity and location of structural cellular proteins [14]. The most noticeable change is the enlargement of atrial cells along with myolysis and the buildup of glycogen around the cell nucleus [14]. These alterations affect atrial contractility and compliance, leading to atrial dilatation [14]. The right atrial remodeling process is characterized by increased collagen deposition, fibrosis, and loss of atrial muscle mass, further contributing to atrial dysfunction [15].

#### 2.1.2 Tricuspid Annular Dilatation

Tricuspid annular dilatation is a key mechanism of AFTR [16]. The tricuspid annulus is a complex, saddle-shaped structure that becomes more planar and circular in patients with AF [17]. This geometric change reduces the leaflet coaptation area and leads to TR [18]. Studies have shown that tricuspid annular diameter is significantly larger in patients with AFTR than those with VFTR [4].

#### 2.1.3 Leaflet Tethering and Malcoaptation

Leaflet tethering and malcoaptation result from AFTR due to atrial dilatation [19]. Furthermore, leaflet tethering and malcoaptation are increasingly observed in VFTR [19]. As the right atrium enlarges and the tricuspid annulus dilates, the tricuspid leaflets stretch and displace apically, leading to incomplete coaptation [20]. This results in a central coaptation gap and significant TR [21].

### 2.2 Comparison with Ventricular Functional TR (VFTR)

#### 2.2.1 Distinct Pathophysiological Mechanisms

While AFTR is primarily driven by right atrial enlargement and dysfunction, VFTR is caused by right ventricular dilatation and dysfunction secondary to left-sided heart disease or pulmonary hypertension [20]. In VFTR, the right ventricle undergoes remodeling and becomes more spherical, leading to tricuspid annular dilatation and leaflet tethering [22]. The right atrium may also enlarge in VFTR, but it is usually a consequence rather than a cause of TR [22].

#### 2.2.2 Different Clinical Implications

**Fig. 2.**
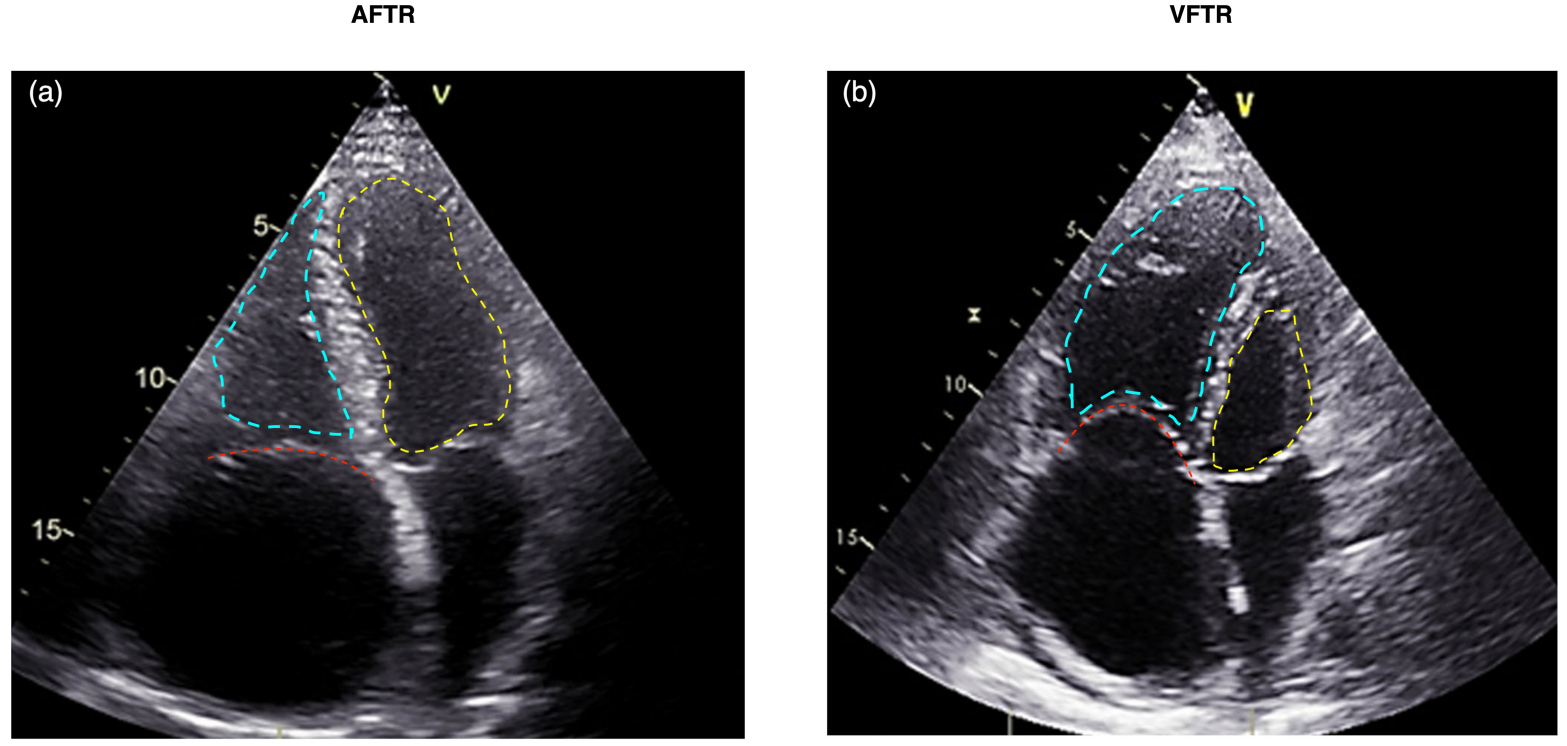
**Echocardiographic comparison of AFTR (a) versus VFTR (b)**. The tricuspid valve is highlighted with a red dashed line, the left ventricle with a yellow dashed line, and the right ventricle with a blue dashed line. (a) Displays AFTR characterized by annular dilatation due to right atrial enlargement, absence of tethering, and a triangular-shaped right ventricle. (b) Illustrates VFTR with a dysfunctional right ventricle where the basal RV diameter exceeds the annular dilatation. AFTR, atrial functional tricuspid regurgitation; VFTR, ventricular functional tricuspid regurgitation; RV, right ventricle.

The distinction between AFTR and VFTR has important clinical implications (Fig. 2) [21]. Patients with AFTR may have a better prognosis than those with VFTR, as the right ventricle is often preserved in AFTR [23]. However, AFTR is associated with increased morbidity and mortality compared to patients without TR [24]. The management strategies for AFTR and VFTR may also differ, with a greater emphasis on rhythm control and right atrial volume reduction in AFTR [3]. Leaflet tethering of more than 10 mm (measured in A4C) is a distinct feature of VFTR [25]. The comparison between AFTR and VFTR is presented in Table 1 (Ref. [3, 20, 21, 22, 25]).

**Table 1.** **Comparison of atrial functional tricuspid regurgitation (AFTR) and ventricular functional tricuspid regurgitation (VFTR)**.

Feature	AFTR	VFTR
Pathophysiology	Right atrial enlargement and dysfunction	Right ventricular dilatation and dysfunction
Common causes	Persistent/permanent atrial fibrillation [20, 21]	Left-sided heart disease, pulmonary hypertension [21, 22]
Diagnostic criteria	Tricuspid annular dilatation, right atrial area enlargement, absence of significant pulmonary hypertension, or left-sided heart disease [20]	Right ventricular dilatation, evidence of pulmonary hypertension, or left-sided heart disease [21]
Leaflet tethering (>10 mm)	Absent [25]	Present [25]
Management strategies	Rhythm control, diuretics, transcatheter interventions [3]	Surgical repair/replacement, transcatheter interventions, management of underlying cause [3]
Prognosis	Variable, depends on successful management of atrial fibrillation and right atrial size reduction [25]	Generally poorer due to underlying heart disease [25]

##  3. Epidemiology and Natural History of AFTR

### 3.1 Prevalence of AFTR in the General Population

AF is the most common sustained cardiac arrhythmia, with a global prevalence of 33.5 million individuals [1]. The prevalence of AFTR in the general population is not well-established, as AFTR is often underdiagnosed and underreported, yet it has been noted that the prevalence of AFTR increases with age and is more common in women than in men [8]. However, studies have shown that the prevalence of TR in patients with AF ranges from 25% to 50% [10]. Moreover, in a community-based study, moderate or severe TR prevalence in patients with AF was 6.5% [26].

### 3.2 Association between AF and TR Severity

There is a strong association between AF and TR severity [10]. Patients with AF have a higher TR prevalence and severity compared to those without AF [8]. In a study by Abe *et al*. [12], the prevalence of moderate or severe TR was significantly higher in patients with AF (25.8%) compared to those with sinus rhythm (15.5%). The severity of TR also correlates with the duration and burden of AF [10]. Patients with persistent or permanent AF have a higher prevalence of severe TR compared to those with paroxysmal AF [8].

### 3.3 Impact of AFTR on Patient Outcomes

#### 3.3.1 Morbidity and Mortality

AFTR is associated with increased morbidity and mortality [8]. Patients with AFTR have a higher risk of heart failure, stroke, and all-cause mortality compared to those without TR; this is mainly due to more pronounced atrial cardiopathy in the later stages of TR [24]. Research by Benfari *et al*. [27] found that the presence of severe TR was associated with a 2-fold increased risk of mortality in patients with heart failure and reduced ejection fraction [21]. The impact of AFTR on mortality is independent of other risk factors, such as age, sex, and left ventricular function [8].

#### 3.3.2 Quality of Life

While echocardiographic and clinical parameters are essential for diagnosing and monitoring AFTR, patient-centered outcomes such as quality of life and functional status are equally important in assessing the impact of the disease and the effectiveness of therapeutic interventions.

Several studies have demonstrated that AFTR is associated with significant impairments in quality of life and functional capacity. In a study by Topilsky *et al*. [8], patients with severe TR reported worse scores on the Minnesota Living with Heart Failure Questionnaire (MLHFQ) compared to those with mild or moderate TR. Similarly, Santoro *et al*. [9] found that patients with severe TR had significantly lower scores on the Short Form-36 (SF-36) questionnaire, indicating reduced physical and mental well-being.

As assessed by the New York Heart Association (NYHA) classification or 6-minute walk test (6MWT), functional status is also impaired in patients with AFTR. In a study by Mehr *et al*. [28], 92% of patients with severe TR were in NYHA class III or IV at baseline, and the mean 6MWT distance was 239 ± 107 meters, indicating significant functional limitation.

### 3.4 Natural History of TR and Right Heart Chamber Remodeling in AF Patients

The natural history of TR and right heart chamber remodeling in patients with AF is not well-defined [7]. However, studies have shown that TR severity tends to progress over time in patients with AF [29]. In a study by Utsunomiya *et al*. [3], the prevalence of severe TR increased from 11% at baseline to 25% after a mean follow-up of 32 months in patients with AF. The progression of TR is associated with ongoing right atrial and ventricular remodeling, characterized by chamber enlargement, dysfunction, and fibrosis [7]. Restoring sinus rhythm through cardioversion or ablation may reduce TR severity and reverse remodeling of the right heart chambers [12].

## 4. Diagnostic Evaluation of AFTR

Accurate diagnosis and assessment of AFTR are crucial for guiding management strategies and predicting patient outcomes. Echocardiography is the primary imaging modality for evaluating AFTR, while other techniques, such as cardiac magnetic resonance imaging (CMR) and computed tomography (CT), can provide complementary information [30]. The key echocardiographic parameters used to assess the severity of AFTR and the cut-off values are presented in Table 2 (Ref. [31, 32]).

**Table 2.** **Echocardiographic parameters for assessing AFTR severity**.

Grades of TR severity	VC [31, 32]	EROA [31, 32]	R vol. [31, 32]
Mild	3 mm	<20 mm^2^	-
Moderate	4.0–6.9 mm	-	-
Severe	≥7.0 mm	-	-
Massive	14–20 mm	60–79 mm^2^	60–74 mL
Torrential	≥21 mm	≥80 mm^2^	≥75 mL

Note: This table summarizes the key echocardiographic parameters used to determine AFTR severity and the cut-off values. Abbreviation: VC, vena contracta; EROA, effective regurgitant orifice area; R vol., regurgitant volume; TR, tricuspid regurgitation; AFTR, atrial functional tricuspid regurgitation.

### 4.1 Echocardiographic Assessment

Echocardiography is the cornerstone of AFTR diagnosis and assessment [28] since it allows for evaluating tricuspid valve morphology, right heart chamber sizes, and the severity of TR. The following key parameters should be assessed during the echocardiographic evaluation of AFTR.

#### 4.1.1 Key Parameters for Assessing TR Severity

4.1.1.1 Grades of TR

Mild TR is defined by an effective regurgitant orifice area (EROA) of <20 mm^2^ and vena contracta (VC) of 3 mm [31]. The severity of TR based on VC width was categorized as moderate (4.0–6.9 mm) or severe (≥7.0 mm) [3]. Massive TR is defined by an effective EROA of 60–79 mm^2^, regurgitant volume (R vol.) of 60–74 mL, and VC of 14–20 mm, while torrential TR is defined by an EROA ≥80 mm^2^, R vol. ≥75 mL, and VC ≥21 mm [32].

4.1.1.2 Tricuspid Annular Diameter

Tricuspid annular dilatation is a hallmark of AFTR [4]. The normal tricuspid annular diameter in adults is 28–35 mm, and values >40 mm are considered significant dilatation [30]. The tricuspid annular diameter should be measured in the apical 4-chamber view at the end-diastole, from the insertion of the septal leaflet to the insertion of the anterior leaflet [33]. Dreyfus *et al*. [4] found that patients with AFTR had significantly larger tricuspid annular diameters than those with ventricular functional TR (43 ± 5 mm *vs*. 37 ± 5 mm).

4.1.1.3 Right Atrial Area

Right atrial enlargement is a key AFTR feature associated with TR severity [3]. The right atrial area should be measured in the apical 4-chamber view at end-systole, tracing the right atrial endocardial border [30]. A right atrial area >18 cm^2^ is considered a significant enlargement [30]. Utsunomiya *et al*. [3] demonstrated that patients with AFTR had significantly larger right atrial areas than the controls (30 ± 10 cm^2^*vs*. 18 ± 5 cm^2^). In disproportionate TR (regurgitation due to structural abnormalities in the tricuspid valve), the right atrial (RA) area and annular dilatation may be underdeveloped. The RA area and tricuspid annular dilatation are important prognostic markers in patients with tricuspid regurgitation, and they provide valuable prognostic information regarding disease severity, risk of heart failure, and overall mortality [34, 35].

4.1.1.4 Right Ventricular Free Wall Longitudinal Strain

Right ventricle (RV) free wall longitudinal strain (RVFWLS) is a sensitive marker of RV dysfunction and can be assessed using speckle-tracking echocardiography [33]. Reduced RVFWLS (fewer negative values) is associated with more severe TR and worse outcomes [36]. In a study by Prihadi *et al*. [7], patients with severe TR had significantly lower RVFWLS than those with mild or moderate TR (–15 ± 5% *vs*. –20 ± 5%).

#### 4.1.2 Role of 3D Echocardiography

Three-dimensional (3D) echocardiography provides unique insights into the complex geometry of the tricuspid valve and right heart chambers in patients with AFTR [37]. Three-dimensional echocardiography allows a more accurate assessment of tricuspid annular size, leaflet morphology, and coaptation defects compared to 2D echocardiography [16]. In a study by Ton-Nu *et al*. [16], 3D echocardiography demonstrated that patients with functional TR had larger tricuspid annular areas, more planar annular shapes, and greater tethering distances than controls. Indeed, 3D echocardiography can also guide interventional procedures for AFTR, such as transcatheter tricuspid valve repair [38].

### 4.2 Other Imaging Modalities

#### 4.2.1 Cardiac Magnetic Resonance Imaging

CMR is a valuable tool for assessing right heart chamber sizes, function, and flow dynamics in patients with AFTR [39, 40]. CMR is considered a gold-standard tool for assessing TR severity and provides a high spatial resolution that can accurately quantify RV volumes and ejection fraction [40]. In a study by Hahn *et al*. [41], CMR-derived RV end-diastolic volume and ejection fraction were independent predictors of mortality in patients with severe TR. CMR can also visualize the tricuspid valve apparatus and identify structural abnormalities [40].

#### 4.2.2 Computed Tomography

CT can provide detailed anatomical information about the tricuspid valve and right heart chambers in patients with AFTR [42, 43]. CT allows for precise measurement of tricuspid annular dimensions, leaflet morphology, and relationship with surrounding structures [43]. This information can be particularly useful for planning surgical or transcatheter interventions for AFTR [42]. In a study by Hinzpeter *et al*. [42], CT-derived tricuspid annular dimensions and leaflet angles predicted procedural success and outcomes after transcatheter tricuspid valve repair.

## 5. AFTR in Specific Patient Populations

AFTR can occur in various clinical settings and patient populations. Thus, understanding the prevalence, mechanisms, and clinical implications of AFTR in these specific groups is essential for tailoring management strategies and improving patient outcomes.

### 5.1 Patients with Atrial Septal Defects

Atrial septal defects (ASDs) are associated with an increased prevalence of AFTR [38]. The left-to-right shunt in ASDs leads to right atrial and ventricular volume overload, which can cause tricuspid annular dilatation and leaflet tethering [44]. In a study by Toyono *et al*. [44], the prevalence of moderate or severe TR in patients with ASDs and chronic AF was significantly higher than in those with ASDs and sinus rhythm. The underlying pathomechanism of TR in ASD patients is complex and involves several factors. The left-to-right shunt increases right heart preload, leading to right atrial and ventricular enlargement. Chronic shunting can lead to pulmonary hypertension, further exacerbating right ventricular dilatation and tricuspid annular enlargement.

The presence of AF in ASD patients can worsen atrial remodeling and contribute to tricuspid annular dilatation. Interestingly, ASD closure can lead to a sudden reduction in right heart preload [45]. This abrupt change in hemodynamics can unmask pre-existing TR or even worsen it in some cases. The mechanism involves reduced right ventricular filling, potentially leading to geometric changes that affect tricuspid valve coaptation, altered right atrial and ventricular compliance due to sudden volume reduction, and possible right ventricular dysfunction in patients with longstanding volume overload [45]. The presence of AFTR in patients with ASDs is associated with worse functional capacity and increased mortality. Surgical or transcatheter closure of ASDs can reduce TR severity and improve right heart chamber sizes and function in many cases. However, persistent AF after ASD closure may limit the reversibility of AFTR and warrant concomitant tricuspid valve intervention [38].

### 5.2 Post-Cardiac Transplantation Patients

AFTR is a common complication after cardiac transplantation, with a reported prevalence of 20–50% [46]. The mechanisms of AFTR in this setting include donor–recipient size mismatch, right ventricular dysfunction, and biatrial anastomosis technique [47]. Biatrial anastomosis, which involves suturing the atria of the donor and recipient together, can lead to atrial enlargement and distorting of the tricuspid valve apparatus [48]. In a study by Wartig *et al*. [46], patients with biatrial anastomosis had a significantly higher prevalence of moderate or severe TR compared to those with bicaval anastomosis (45% *vs*. 15%); TR occurs due to structural changes in the atrial chambers. The presence of AFTR after cardiac transplantation is associated with reduced exercise capacity, right ventricular dysfunction, and increased mortality [46]. Management strategies for AFTR in this population include diuretics, pulmonary vasodilators, and tricuspid valve intervention in selected cases [47].

### 5.3 Patients after Left-Sided Valve Surgery

AFTR can develop or worsen after left-sided valve surgery, particularly in patients with pre-existing AF [49]. The mechanisms of AFTR in this setting include right ventricular dysfunction due to cardiopulmonary bypass, pericardial constraint, and progression of underlying atrial and valvular disease [50]. In a study by Dreyfus *et al*. [50], the prevalence of moderate or severe TR increased from 27% preoperatively to 68% at 5 years after mitral valve surgery in patients with pre-existing AF. The presence of AFTR after left-sided valve surgery is associated with reduced functional capacity, right ventricular dysfunction, and increased mortality [51]. Management strategies for AFTR in this population include aggressive treatment of AF, optimization of medical therapy, and consideration of concomitant or staged tricuspid valve intervention [52, 53, 54, 55, 56]. In a study by Chikwe *et al*. [52], concomitant tricuspid valve repair during left-sided valve surgery was associated with improved long-term survival and reduced TR progression compared to left-sided valve surgery alone.

## 6. Management Strategies for AFTR

Managing AFTR is challenging and requires a multidisciplinary approach tailored to the individual patient. Treatment options include medical therapy, surgical interventions, and percutaneous procedures. The choice of intervention depends on various factors, including TR severity, underlying cardiac conditions, patient characteristics, and institutional expertise [57]. The indications, advantages, disadvantages, and outcomes of surgical and percutaneous interventions for AFTR are presented in Table 3 (Ref. [58, 59, 60, 61, 62]).

**Table 3.** **Comparison of surgical and percutaneous interventions for AFTR**.

Characteristic	Surgical interventions	Percutaneous procedures
Indications	Concomitant left-sided valve disease and acceptable surgical risk [58]	High surgical risk, isolated TR, and prior cardiac surgery [61]
Advantages	Definitive repair or replacement and concomitant procedures possible [59]	Less invasive and shorter recovery time [62]
Disadvantages	Higher perioperative risk and longer recovery time [60]	Limited long-term data and a potential need for reintervention [62]
Outcomes	Improved symptoms and survival, but significant morbidity and mortality [60]	Promising early results [62], but long-term durability unknown

Note: This table compares the indications, advantages, disadvantages, and outcomes of surgical and percutaneous interventions for AFTR. Abbreviation: TR, tricuspid regurgitation; AFTR, atrial functional tricuspid regurgitation.

### 6.1 Medical Therapy

#### 6.1.1 Diuretics and Salt Restriction

Diuretics and salt restriction are the mainstay of medical therapy for patients with AFTR and right heart failure symptoms [57]. Loop diuretics, such as furosemide, can help reduce peripheral edema and improve functional capacity [63]. However, aggressive diuresis may lead to renal dysfunction and electrolyte abnormalities, requiring careful monitoring [63].

#### 6.1.2 Rhythm Control Strategies

Rhythm control strategies, including antiarrhythmic drugs and catheter ablation, may benefit patients with AFTR [12]. Restoration and maintenance of sinus rhythm can improve right atrial and ventricular function, potentially reducing TR severity [12]. In a study by Abe *et al*. [12], successful catheter ablation for AF was associated with a significant reduction in TR severity and improvement in right ventricular function. A study by Soulat-Dufour *et al*. [64] found that actively restoring sinus rhythm (SR) by cardioversion and/or ablation is connected with reduced functional atrioventricular regurgitation.

### 6.2 Surgical Interventions

#### 6.2.1 Indications and Timings

Current guidelines recommend concomitant tricuspid valve repair for patients with severe TR (stages C and D) undergoing left-sided valve surgery [58]. However, there is growing evidence to support earlier intervention in patients with moderate TR (stage B) and tricuspid annular dilation (>40 mm) to prevent disease progression [50]. Dreyfus *et al*. [50] proposed a more aggressive approach, suggesting that tricuspid annuloplasty should be performed in patients with a tricuspid annular diameter >40 mm, regardless of TR severity.

#### 6.2.2 Techniques and Outcomes

Surgical techniques for AFTR include tricuspid valve repair (annuloplasty) and replacement. Ring annuloplasty is preferred for suitable valve morphology, offering lower operative mortality and better long-term outcomes than replacements [59]. A meta-analysis by Veen *et al*. [49] showed ring annuloplasty associated with lower recurrent TR risk and improved survival versus suture annuloplasty. However, for tricuspid valve replacement, a recent meta-analysis by Scotti *et al*. [60] revealed relatively poor outcomes, with 12% operative mortality and frequent complications. Long-term outcomes for bioprosthetic TVR showed incidence rates of 6 per 100 person-years for death and 8 per 100 person-years for significant TR recurrence. Notably, this analysis did not differentiate between AFTR and VFTR, highlighting the need for future studies to address this distinction [60]. In a study by Gammie *et al*. [65], it was found that adding concomitant tricuspid annuloplasty (TA) during mitral valve repair (MVR) reduced the rate of treatment failure at 2 years compared to MVR alone. Treatment failure was defined as the composite of death, re-operation for tricuspid regurgitation, progression of TR by two grades from baseline, or the presence of severe TR at 2 years [65]. However, this reduction in TR progression was associated with a higher risk of permanent pacemaker implantation [65].

### 6.3 Percutaneous Procedures

#### 6.3.1 Edge-to-Edge Repair

**Fig. 3.**
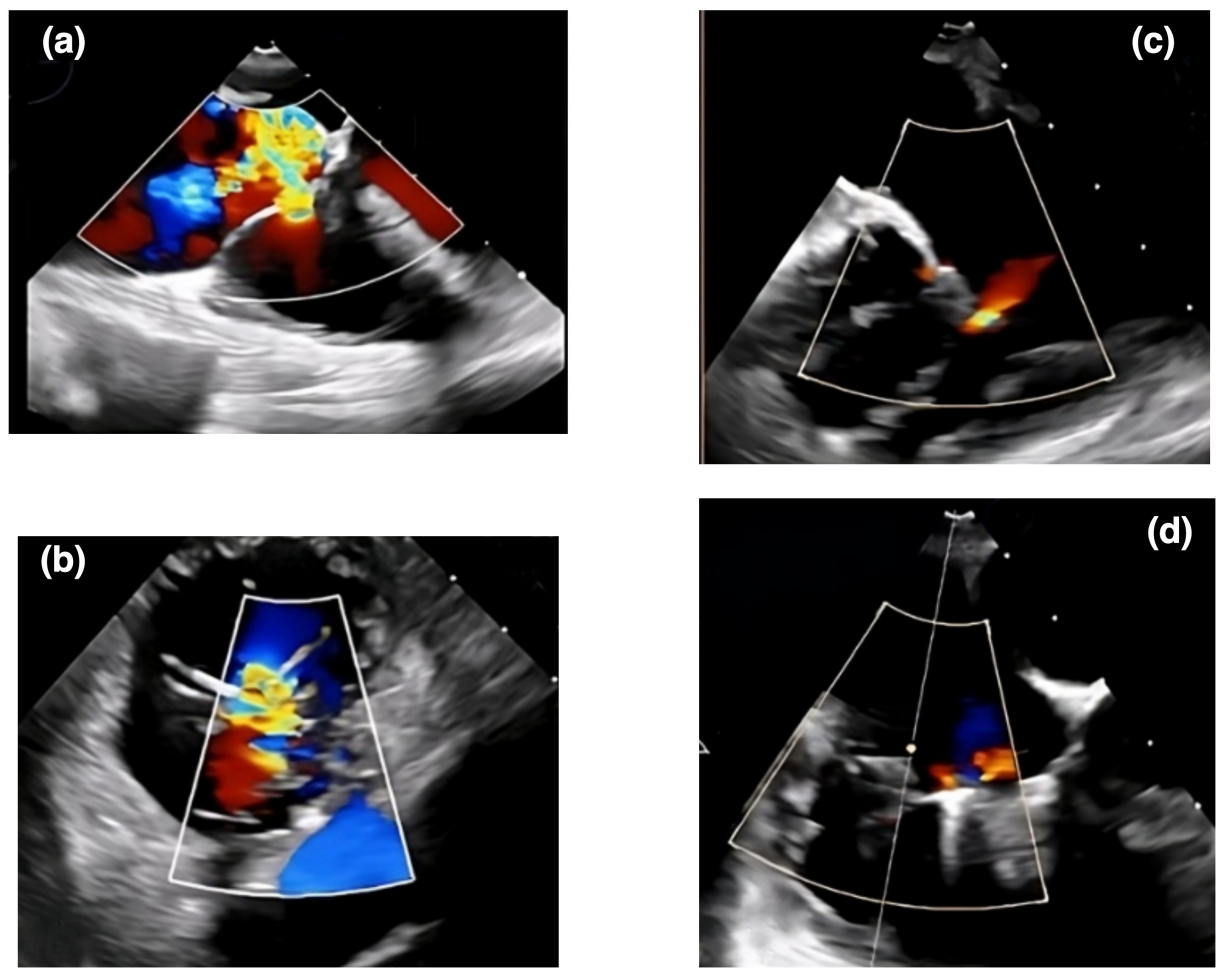
**An echocardiographic representation of a transcatheter therapy outcome using the TriClip system for AFTR**. Panels (a) and (b) show Doppler echocardiographic images of tricuspid regurgitation before a TriClip intervention. Panels (c) and (d) display echocardiographic views of tricuspid regurgitation after the TriClip intervention. AFTR, atrial functional tricuspid regurgitation.

Transcatheter edge-to-edge repair using the MitraClip system (Abbott Vascular, Santa Clara, CA, USA) has emerged as a promising treatment option for patients with AFTR at high surgical risk [66]. The TRILUMINATE trial demonstrated significant improvements in TR grade, functional status, and quality of life 1 year after the procedure [67]. However, the long-term durability and impact on clinical outcomes remain to be established. In a propensity-matched analysis by Taramasso *et al*. [66], transcatheter edge-to-edge repair was associated with similar improvements in TR severity and functional status compared to medical therapy but with a higher rate of major adverse events. To accurately assess the severity and mechanisms of tricuspid regurgitation, it is crucial to employ detailed transthoracic and transesophageal echocardiograms to evaluate the tricuspid valve anatomy and effectively guide patient selection for transcatheter edge-to-edge repair [68]. For tricuspid transcatheter edge-to-edge repair (T-TEER), the TriClip and PASCAL systems are designated therapies with specific features (Fig. 3). TriClip uses a right heart-specific guide and delivery system, available in various clip sizes with independent gripper action and an active locking mechanism [69]. PASCAL offers high maneuverability and independent leaflet capture capability [70]. Both systems have received regulatory approval and are used to treat severe tricuspid regurgitation. Off-label usage of the MitraClip system for tricuspid repair should only be considered in countries where both TriClip and PASCAL are unavailable, ensuring focus on devices specifically designed for tricuspid valve intervention [71]. Future studies should continue to evaluate the safety, efficacy, and durability of these technologies compared to surgical and medical therapies [5, 57].

#### 6.3.2 Transcatheter Tricuspid Valve Replacement

Transcatheter tricuspid valve replacement (TTVR) is an emerging technology for treating AFTR in patients with anatomical challenges or failed previous repairs [61]. Early feasibility studies have shown promising results, with high procedural success rates and significant improvements in TR severity and functional status [61]. The EVOQUE tricuspid valve replacement system (Edwards Lifesciences) has advanced beyond early feasibility studies and received approval in 2022, making it available for clinical use in Europe. A study by Webb *et al*. [72] reported favorable 1-year outcomes using the EVOQUE system, including a high technical success rate, significant reduction in TR severity, and improved functional status. At 1 year, 96% of patients had TR grade ≤2+, and 70% were in NYHA functional class I/II, with a low all-cause mortality rate of 7%. Another system showing promising results is the TricValve Transcatheter Bicaval Valves System, designed for patients with severe TR and caval reflux [72]. Blasco-Turrión* et al*. [73] recently reported successful implantation in 97% of cases, with significant improvements in the New York Heart Association functional class and quality of life measures at 30 days post-procedure. These advancements represent important progress in TTVR technology [73]. However, ongoing research and larger clinical trials are still needed to further evaluate the long-term safety, efficacy, and durability of TTVR in patients with AFTR.

#### 6.3.3 Transcatheter Annuloplasty

Transcatheter annuloplasty (Cardioband), orthotopic, and heterotopic valve replacement are preferred methods for treating patients with AFTR due to their association with lower operative mortality and better long-term outcomes [62]. These procedures rely on crucial pre-procedure imaging techniques, such as cardiac computed tomography (CCT) and vascular computed tomography angiography (CTA), which provide meticulous anatomical assessments and accurate evaluations of annular size [62, 74]. Specifically, cardiac computed tomography angiography (CCTA) is used before transcatheter annuloplasty (Cardioband) to assess the structural and size characteristics of the tricuspid annulus [75]. CCTA also evaluates the distance from coronary vessels, the catheter’s insertion, the expected angle from the inferior vena cava to the right atrium, and its alignment with the tricuspid annulus. Additionally, CCTA provides the necessary angulations for fluoroscopy during implantation [75]. Several devices are available in clinical practice, including coaptation devices and annuloplasty systems, such as the Cardioband and heterotopic valve implantation techniques; however, newer generations and innovative technologies are in development [76, 77]. The Cardioband Tricuspid System is specifically designed to reduce the size of the tricuspid annulus, improving leaflet coaptation and reducing regurgitation [69].

### 6.4 Comparative Effectiveness and Clinical Algorithms

Comparative studies of surgical and percutaneous interventions for AFTR are limited, and the optimal treatment strategy remains unclear. In a meta-analysis by Veen *et al*. [49], surgical tricuspid valve repair was associated with lower rates of recurrent TR and improved survival compared to percutaneous interventions. However, this analysis included mostly observational studies and was limited by heterogeneity in patient populations and treatment techniques.

Recent comparative studies have provided valuable insights into the outcomes of transcatheter tricuspid valve interventions in AFTR and VFTR patients. A study by Russo *et al*. [78] compared outcomes of T-TEER in AFTR and VFTR patients, finding similar procedural outcomes for both groups, with differences in mortality primarily attributed to underlying diseases. For transcatheter annuloplasty, Barbieri *et al*. [79] compared the procedural success of the Cardioband device in AFTR and VFTR patients, finding no significant differences between groups in annulus diameter reduction, vena contracta reduction, or effective regurgitation orifice area reduction. Improvement in TR severity of at least two grades was similar in both groups (90.0% in VFTR *vs*. 91.4% in AFTR) [79]. These studies demonstrate that both T-TEER and transcatheter annuloplasty can effectively treat both forms of functional TR with similar procedural success rates. Furthermore, they highlight that differences in long-term outcomes may be more related to underlying cardiac conditions than the type of functional TR.

### 6.5 Prognostic Implications of AFTR vs. VFTR

Patients with AFTR demonstrated significantly better long-term survival than those with VFTR. The 10-year cumulative survival rate for AFTR was 78%, whereas it was 46% for VFTR (*p* < 0.001) [80]. This survival advantage persisted even after adjusting for relevant clinical and echocardiographic variables. Multivariable Cox regression analysis revealed that VFTR was independently associated with worse overall survival than AFTR (HR: 2.292, *p* < 0.001), which held true for all VFTR subtypes, including left-sided cardiac disease, pulmonary hypertension, and right ventricular dysfunction. Echocardiographic differences between the two groups may contribute to this prognostic disparity [80]. AFTR patients presented with smaller right ventricular dimensions, larger tricuspid valve annular diameter, larger maximal right atrial dimensions/volumes, and less leaflet tenting than VFTR patients. These findings underscore the importance of distinguishing between AFTR and VFTR in clinical practice and research, as they have significant implications for patient prognosis and management strategies.

## 7. Future Directions and Research Opportunities

Despite the growing recognition of AFTR as a distinct entity with significant clinical implications, several gaps in our understanding of this condition still need to be addressed. Thus, future research efforts should address these knowledge gaps and explore novel therapeutic strategies to improve patient outcomes.

### 7.1 Gaps in the Current Understanding of AFTR

One of the major limitations in our current understanding of AFTR is the lack of standardized diagnostic criteria and severity grading [57]. The current guidelines for assessing TR severity were developed primarily for patients with left-sided heart disease and may not accurately reflect the unique pathophysiology of AFTR [28, 30]. Future studies should aim to establish specific diagnostic criteria and severity grading systems for AFTR, considering the complex interplay between atrial and right ventricular remodeling [3, 7].

Another gap in our knowledge is the natural history and prognostic implications of AFTR [8, 21]. While several studies have demonstrated an association between AFTR and adverse outcomes, the long-term trajectory of this condition and its impact on patient survival and quality of life remain poorly defined [8, 9]. Future research should focus on elucidating the natural history of AFTR and identifying prognostic markers to guide risk stratification and treatment decisions [8, 28].

### 7.2 Need for Prospective Studies

#### 7.2.1 Natural History

Prospective, longitudinal studies are needed to better characterize the natural history of AFTR and its progression over time [29]. These studies should include patients with varying degrees of AFTR severity and assess the impact of clinical factors, such as AF burden, right ventricular function, and pulmonary hypertension, on the course of the disease [81]. Serial echocardiographic assessments and biomarker measurements could provide valuable insights into the mechanisms and predictors of AFTR progression [3, 28].

#### 7.2.2 Optimal Management Strategies

There is a lack of data on the optimal management strategies for patients with AFTR [57]. Current treatment approaches are largely extrapolated from studies of patients with left-sided heart disease and may not be directly applicable to the AFTR population [49, 63]. Prospective, randomized trials are needed to evaluate the efficacy and safety of various therapeutic interventions, such as diuretics, pulmonary vasodilators, and rhythm control strategies, in patients with AFTR [63, 82]. These studies should also assess the impact of different management strategies on patient-centered outcomes, such as functional capacity and quality of life [28, 83].

#### 7.2.3 Long-Term Outcomes

Long-term outcome data are essential for guiding clinical decision-making and patient counseling in the setting of AFTR [8, 22]. Prospective studies with extended follow-up periods are needed to evaluate the impact of AFTR on patient survival, cardiovascular events, and healthcare utilization [29, 84]. These studies should also assess the long-term durability and effectiveness of various therapeutic interventions, such as transcatheter tricuspid valve repair or replacement [66, 67].

### 7.3 Potential Novel Therapeutic Targets and Interventions

As our understanding of the pathophysiology of AFTR continues to evolve, novel therapeutic targets and interventions may emerge [85]. For example, targeting the renin–angiotensin–aldosterone system (RAAS) with pharmacologic agents, such as angiotensin-converting enzyme inhibitors or aldosterone antagonists, may help to attenuate right atrial and ventricular remodeling in patients with AFTR [10]. Similarly, novel antifibrotic therapies, such as pirfenidone or nintedanib, may have a role in preventing or reversing the structural changes associated with AFTR [86].

## 8. Conclusions

AFTR is a clinically significant condition with distinct pathophysiology, epidemiology, and management strategies. This review underscores the necessity of accurate diagnosis, timely intervention, and personalized treatment approaches since raising awareness among healthcare professionals is crucial for enhancing patient care and outcomes. Despite advancements, several important gaps in our understanding of AFTR remain, warranting further investigation. Thus, prospective, longitudinal studies are needed to improve the characterization of the natural history, optimal management strategies, and long-term outcomes of this condition. Therefore, randomized controlled trials for each distinct form of TR are essential to establish evidence-based treatment approaches. Moreover, novel therapeutic targets and interventions, such as RAAS inhibition, antifibrotic therapies, and innovative transcatheter devices, should be explored in future research efforts. By addressing these knowledge gaps and advancing the field of AFTR, we can significantly improve the care and outcomes of patients with these increasingly recognized and clinically significant conditions.

## References

[1] Chugh SS, Havmoeller R, Narayanan K, Singh D, Rienstra M, Benjamin EJ (2014). Worldwide epidemiology of atrial fibrillation: a Global Burden of Disease 2010 Study. *Circulation*.

[2] Odutayo A, Wong CX, Hsiao AJ, Hopewell S, Altman DG, Emdin CA (2016). Atrial fibrillation and risks of cardiovascular disease, renal disease, and death: systematic review and meta-analysis. *BMJ (Clinical Research Ed.)*.

[3] Utsunomiya H, Itabashi Y, Mihara H, Berdejo J, Kobayashi S, Siegel RJ (2017). Functional Tricuspid Regurgitation Caused by Chronic Atrial Fibrillation: A Real-Time 3-Dimensional Transesophageal Echocardiography Study. *Circulation. Cardiovascular Imaging*.

[4] Dreyfus J, Durand-Viel G, Raffoul R, Alkhoder S, Hvass U, Radu C (2015). Comparison of 2-Dimensional, 3-Dimensional, and Surgical Measurements of the Tricuspid Annulus Size: Clinical Implications. *Circulation. Cardiovascular Imaging*.

[5] Hahn RT (2016). State-of-the-Art Review of Echocardiographic Imaging in the Evaluation and Treatment of Functional Tricuspid Regurgitation. *Circulation. Cardiovascular Imaging*.

[6] Guérin A, Dreyfus J, Le Tourneau T, Sportouch C, Lairez O, Eicher JC (2019). Secondary tricuspid regurgitation: Do we understand what we would like to treat?. *Archives of Cardiovascular Diseases*.

[7] Prihadi EA, Delgado V, Leon MB, Enriquez-Sarano M, Topilsky Y, Bax JJ (2019). Morphologic Types of Tricuspid Regurgitation: Characteristics and Prognostic Implications. *JACC. Cardiovascular Imaging*.

[8] Topilsky Y, Maltais S, Medina Inojosa J, Oguz D, Michelena H, Maalouf J (2019). Burden of Tricuspid Regurgitation in Patients Diagnosed in the Community Setting. *JACC. Cardiovascular Imaging*.

[9] Santoro C, Marco Del Castillo A, González-Gómez A, Monteagudo JM, Hinojar R, Lorente A (2019). Mid-term outcome of severe tricuspid regurgitation: are there any differences according to mechanism and severity? European Heart Journal. *Cardiovascular Imaging*.

[10] Deferm S, Bertrand PB, Verbrugge FH, Verhaert D, Rega F, Thomas JD (2019). Atrial Functional Mitral Regurgitation: JACC Review Topic of the Week. *Journal of the American College of Cardiology*.

[11] Kim YS, Jeong HG, Hwang IC, Kim BJ, Kwon JM, Bae HJ (2023). Tricuspid regurgitation: a hidden risk factor for atrial fibrillation related stroke?. *Frontiers in Cardiovascular Medicine*.

[12] Abe Y, Akamatsu K, Ito K, Matsumura Y, Shimeno K, Naruko T (2018). Prevalence and Prognostic Significance of Functional Mitral and Tricuspid Regurgitation Despite Preserved Left Ventricular Ejection Fraction in Atrial Fibrillation Patients. *Circulation Journal*.

[13] Sanfilippo AJ, Abascal VM, Sheehan M, Oertel LB, Harrigan P, Hughes RA (1990). Atrial enlargement as a consequence of atrial fibrillation. A prospective echocardiographic study. *Circulation*.

[14] Allessie M, Ausma J, Schotten U (2002). Electrical, contractile and structural remodeling during atrial fibrillation. *Cardiovascular Research*.

[15] Yamamoto Y, Daimon M, Nakanishi K, Nakao T, Hirokawa M, Ishiwata J (2022). Incidence of atrial functional tricuspid regurgitation and its correlation with tricuspid valvular deformation in patients with persistent atrial fibrillation. *Frontiers in Cardiovascular Medicine*.

[16] Ton-Nu TT, Levine RA, Handschumacher MD, Dorer DJ, Yosefy C, Fan D (2006). Geometric determinants of functional tricuspid regurgitation: insights from 3-dimensional echocardiography. *Circulation*.

[17] Fukuda S, Saracino G, Matsumura Y, Daimon M, Tran H, Greenberg NL (2006). Three-dimensional geometry of the tricuspid annulus in healthy subjects and in patients with functional tricuspid regurgitation: a real-time, 3-dimensional echocardiographic study. *Circulation*.

[18] Spinner EM, Shannon P, Buice D, Jimenez JH, Veledar E, Del Nido PJ (2011). In vitro characterization of the mechanisms responsible for functional tricuspid regurgitation. *Circulation*.

[19] Muraru D, Badano LP, Hahn RT, Lang RM, Delgado V, Wunderlich NC (2024). Atrial secondary tricuspid regurgitation: pathophysiology, definition, diagnosis, and treatment. *European Heart Journal*.

[20] Topilsky Y, Khanna A, Le Tourneau T, Park S, Michelena H, Suri R (2012). Clinical context and mechanism of functional tricuspid regurgitation in patients with and without pulmonary hypertension. *Circulation. Cardiovascular Imaging*.

[21] Mutlak D, Lessick J, Reisner SA, Aronson D, Dabbah S, Agmon Y (2007). Echocardiography-based spectrum of severe tricuspid regurgitation: the frequency of apparently idiopathic tricuspid regurgitation. *Journal of the American Society of Echocardiography*.

[22] Shiran A, Sagie A (2009). Tricuspid regurgitation in mitral valve disease incidence, prognostic implications, mechanism, and management. *Journal of the American College of Cardiology*.

[23] Topilsky Y, Khanna AD, Oh JK, Nishimura RA, Enriquez-Sarano M, Jeon YB (2011). Preoperative factors associated with adverse outcome after tricuspid valve replacement. *Circulation*.

[24] Nath J, Foster E, Heidenreich PA (2004). Impact of tricuspid regurgitation on long-term survival. *Journal of the American College of Cardiology*.

[25] Schlotter F, Dietz MF, Stolz L, Kresoja KP, Besler C, Sannino A (2022). Atrial Functional Tricuspid Regurgitation: Novel Definition and Impact on Prognosis. *Circulation. Cardiovascular Interventions*.

[26] Singh JP, Evans JC, Levy D, Larson MG, Freed LA, Fuller DL (1999). Prevalence and clinical determinants of mitral, tricuspid, and aortic regurgitation (the Framingham Heart Study). *The American Journal of Cardiology*.

[27] Benfari G, Antoine C, Miller WL, Thapa P, Topilsky Y, Rossi A (2019). Excess Mortality Associated With Functional Tricuspid Regurgitation Complicating Heart Failure With Reduced Ejection Fraction. *Circulation*.

[28] Mehr M, Taramasso M, Besler C, Ruf T, Connelly KA, Weber M (2019). 1-Year Outcomes After Edge-to-Edge Valve Repair for Symptomatic Tricuspid Regurgitation: Results From the TriValve Registry. *JACC. Cardiovascular Interventions*.

[29] Shiran A, Najjar R, Adawi S, Aronson D (2014). Risk factors for progression of functional tricuspid regurgitation. *The American Journal of Cardiology*.

[30] Lang RM, Badano LP, Mor-Avi V, Afilalo J, Armstrong A, Ernande L (2015). Recommendations for cardiac chamber quantification by echocardiography in adults: an update from the American Society of Echocardiography and the European Association of Cardiovascular Imaging. *Journal of the American Society of Echocardiography*.

[31] Hahn RT, Zamorano JL (2017). The need for a new tricuspid regurgitation grading scheme. *European Heart Journal. Cardiovascular Imaging*.

[32] Go YY, Dulgheru R, Lancellotti P (2018). The Conundrum of Tricuspid Regurgitation Grading. *Frontiers in Cardiovascular Medicine*.

[33] Rudski LG, Lai WW, Afilalo J, Hua L, Handschumacher MD, Chandrasekaran K (2010). Guidelines for the echocardiographic assessment of the right heart in adults: a report from the American Society of Echocardiography endorsed by the European Association of Echocardiography, a registered branch of the European Society of Cardiology, and the Canadian Society of Echocardiography. *Journal of the American Society of Echocardiography*.

[34] Henning RJ (2022). Tricuspid valve regurgitation: current diagnosis and treatment. *American Journal of Cardiovascular Disease*.

[35] Fortuni F, Dietz MF, Prihadi EA, van der Bijl P, De Ferrari GM, Bax JJ (2021). Ratio between Vena Contracta Width and Tricuspid Annular Diameter: Prognostic Value in Secondary Tricuspid Regurgitation. *Journal of the American Society of Echocardiography*.

[36] Dietz MF, Prihadi EA, van der Bijl P, Goedemans L, Mertens BJA, Gursoy E (2019). Prognostic Implications of Right Ventricular Remodeling and Function in Patients With Significant Secondary Tricuspid Regurgitation. *Circulation*.

[37] Muraru D, Hahn RT, Soliman OI, Faletra FF, Basso C, Badano LP (2019). 3-Dimensional Echocardiography in Imaging the Tricuspid Valve. *JACC. Cardiovascular Imaging*.

[38] Hahn RT, Nabauer M, Zuber M, Nazif TM, Hausleiter J, Taramasso M (2019). Intraprocedural Imaging of Transcatheter Tricuspid Valve Interventions. *JACC. Cardiovascular Imaging*.

[39] Ahn Y, Koo HJ, Kang JW, Yang DH (2021). Tricuspid Valve Imaging and Right Ventricular Function Analysis Using Cardiac CT and MRI. *Korean Journal of Radiology*.

[40] Gulsin GS, Singh A, McCann GP (2017). Cardiovascular magnetic resonance in the evaluation of heart valve disease. *BMC Medical Imaging*.

[41] Hahn RT, Thomas JD, Khalique OK, Cavalcante JL, Praz F, Zoghbi WA (2019). Imaging Assessment of Tricuspid Regurgitation Severity. *JACC. Cardiovascular Imaging*.

[42] Hinzpeter R, Eberhard M, Burghard P, Tanner FC, Taramasso M, Manka R (2017). Computed tomography in patients with tricuspid regurgitation prior to transcatheter valve repair: dynamic analysis of the annulus with an individually tailored contrast media protocol. *EuroIntervention*.

[43] Praz F, Khalique OK, Dos Reis Macedo LG, Pulerwitz TC, Jantz J, Wu IY (2018). Comparison between Three-Dimensional Echocardiography and Computed Tomography for Comprehensive Tricuspid Annulus and Valve Assessment in Severe Tricuspid Regurgitation: Implications for Tricuspid Regurgitation Grading and Transcatheter Therapies. *Journal of the American Society of Echocardiography*.

[44] Toyono M, Krasuski RA, Pettersson GB, Matsumura Y, Yamano T, Shiota T (2009). Persistent tricuspid regurgitation and its predictor in adults after percutaneous and isolated surgical closure of secundum atrial septal defect. *The American Journal of Cardiology*.

[45] Takaya Y, Akagi T, Kijima Y, Nakagawa K, Ito H (2017). Functional Tricuspid Regurgitation After Transcatheter Closure of Atrial Septal Defect in Adult Patients: Long-Term Follow-Up. *JACC. Cardiovascular Interventions*.

[46] Wartig M, Tesan S, Gäbel J, Jeppsson A, Selimovic N, Holmberg E (2014). Tricuspid regurgitation influences outcome after heart transplantation. *The Journal of Heart and Lung Transplantation*.

[47] Kwon MH, Shemin RJ (2017). Tricuspid valve regurgitation after heart transplantation. *Annals of Cardiothoracic Surgery*.

[48] Fiorelli AI, Stolf NAG, Abreu Filho CAC, Santos RHB, Buco FHA, Fiorelli LR (2007). Prophylactic donor tricuspid annuloplasty in orthotopic bicaval heart transplantation. *Transplantation Proceedings*.

[49] Veen KM, Etnel JRG, Quanjel TJM, Mokhles MM, Huygens SA, Rasheed M (2020). Outcomes after surgery for functional tricuspid regurgitation: a systematic review and meta-analysis. *European Heart Journal. Quality of Care & Clinical Outcomes*.

[50] Dreyfus GD, Corbi PJ, Chan KMJ, Bahrami T (2005). Secondary tricuspid regurgitation or dilatation: which should be the criteria for surgical repair. *The Annals of Thoracic Surgery*.

[51] Kwak JJ, Kim YJ, Kim MK, Kim HK, Park JS, Kim KH (2008). Development of tricuspid regurgitation late after left-sided valve surgery: a single-center experience with long-term echocardiographic examinations. *American Heart Journal*.

[52] Chikwe J, Itagaki S, Anyanwu A, Adams DH (2015). Impact of Concomitant Tricuspid Annuloplasty on Tricuspid Regurgitation, Right Ventricular Function, and Pulmonary Artery Hypertension After Repair of Mitral Valve Prolapse. *Journal of the American College of Cardiology*.

[53] Pfannmüller B, Misfeld M, Borger MA, Etz CD, Funkat AK, Garbade J (2012). Isolated reoperative minimally invasive tricuspid valve operations. *The Annals of Thoracic Surgery*.

[54] Ro SK, Kim JB, Jung SH, Choo SJ, Chung CH, Lee JW (2013). Mild-to-moderate functional tricuspid regurgitation in patients undergoing mitral valve surgery. *The Journal of Thoracic and Cardiovascular Surgery*.

[55] Chen Z, Ke Y, Xie X, Huang J, Zeng Q, Guo H (2019). Beating-Heart Totally Endoscopic Tricuspid Valvuloplasty in Reoperative Cardiac Surgery. *The Annals of Thoracic Surgery*.

[56] Chen J, Cheng Z, Dong N, Dong L, Guo H, Guo Y (2023). 2022 CMICS Expert Consensus on the Management of Isolated Tricuspid Regurgitation after Left-Sided Valve Surgery. *Reviews in Cardiovascular Medicine*.

[57] Antunes MJ, Rodríguez-Palomares J, Prendergast B, De Bonis M, Rosenhek R, Al-Attar N (2017). Management of tricuspid valve regurgitation: Position statement of the European Society of Cardiology Working Groups of Cardiovascular Surgery and Valvular Heart Disease. *European Journal of Cardio-Thoracic Surgery*.

[58] Vahanian A, Beyersdorf F, Praz F, Milojevic M, Baldus S, Bauersachs J (2022). 2021 ESC/EACTS Guidelines for the management of valvular heart disease. *European Heart Journal*.

[59] Zack CJ, Fender EA, Chandrashekar P, Reddy YNV, Bennett CE, Stulak JM (2017). National Trends and Outcomes in Isolated Tricuspid Valve Surgery. *Journal of the American College of Cardiology*.

[60] Scotti A, Sturla M, Granada JF, Kodali SK, Coisne A, Mangieri A (2022). Outcomes of isolated tricuspid valve replacement: a systematic review and meta-analysis of 5,316 patients from 35 studies. *EuroIntervention*.

[61] Kodali SK, Hahn RT, Davidson CJ, Narang A, Greenbaum A, Gleason P (2023). 1-Year Outcomes of Transcatheter Tricuspid Valve Repair. *Journal of the American College of Cardiology*.

[62] Blusztein DI, Hahn RT (2023). New therapeutic approach for tricuspid regurgitation: Transcatheter tricuspid valve replacement or repair. *Frontiers in Cardiovascular Medicine*.

[63] Maeder MT, Holst DP, Kaye DM (2008). Tricuspid regurgitation contributes to renal dysfunction in patients with heart failure. *Journal of Cardiac Failure*.

[64] Soulat-Dufour L, Lang S, Addetia K, Ederhy S, Adavane-Scheuble S, Chauvet-Droit M (2022). Restoring Sinus Rhythm Reverses Cardiac Remodeling and Reduces Valvular Regurgitation in Patients With Atrial Fibrillation. *Journal of the American College of Cardiology*.

[65] Gammie JS, Chu MWA, Falk V, Overbey JR, Moskowitz AJ, Gillinov M (2022). Concomitant Tricuspid Repair in Patients with Degenerative Mitral Regurgitation. *The New England Journal of Medicine*.

[66] Taramasso M, Benfari G, van der Bijl P, Alessandrini H, Attinger-Toller A, Biasco L (2019). Transcatheter Versus Medical Treatment of Patients With Symptomatic Severe Tricuspid Regurgitation. *Journal of the American College of Cardiology*.

[67] Nickenig G, Weber M, Lurz P, von Bardeleben RS, Sitges M, Sorajja P (2019). Transcatheter edge-to-edge repair for reduction of tricuspid regurgitation: 6-month outcomes of the TRILUMINATE single-arm study. *Lancet*.

[68] So CY, Fan Y, Su M, Wang Y, He B, Lee APW (2023). Tricuspid Transcatheter Edge-to-edge Repair. *JAPSC*.

[69] Madhavan MV, Agarwal V, Hahn RT (2024). Transcatheter Therapy for the Tricuspid Valve: A Focused Review of Edge-to-Edge Repair and Orthotopic Valve Replacement. *Current Cardiology Reports*.

[70] Godoy Rivas C, Agarwal V, Tomlinson S, Lebehn M, Kodali S, Hahn RT (2023). Tricuspid Leaflet Gap-Reduction Maneuvers During Transcatheter Tricuspid Valve Repair. *JACC. Case Reports*.

[71] Ruf TF, Hahn RT, Kreidel F, Beiras-Fernandez A, Hell M, Gerdes P (2021). Short-Term Clinical Outcomes of Transcatheter Tricuspid Valve Repair With the Third-Generation MitraClip XTR System. *JACC. Cardiovascular Interventions*.

[72] Webb JG, Chuang AMY, Meier D, von Bardeleben RS, Kodali SK, Smith RL (2022). Transcatheter Tricuspid Valve Replacement With the EVOQUE System: 1-Year Outcomes of a Multicenter, First-in-Human Experience. *JACC. Cardiovascular Interventions*.

[73] Blasco-Turrión S, Briedis K, Estévez-Loureiro R, Sánchez-Recalde A, Cruz-González I, Pascual I (2024). Bicaval TricValve Implantation in Patients With Severe Symptomatic Tricuspid Regurgitation: 1-Year Follow-Up Outcomes. *JACC. Cardiovascular Interventions*.

[74] Pulerwitz TC, Khalique OK, Leb J, Hahn RT, Nazif TM, Leon MB (2020). Optimizing Cardiac CT Protocols for Comprehensive Acquisition Prior to Percutaneous MV and TV Repair/Replacement. *JACC. Cardiovascular Imaging*.

[75] Barbieri F, Niehues SM, Feuchtner GM, Skurk C, Landmesser U, Polak-Krasna K (2024). Cardiac Computed Tomography Screening for Tricuspid Transcatheter Annuloplasty Implantation. *Circulation. Cardiovascular Imaging*.

[76] Braun D, Rommel KP, Orban M, Karam N, Brinkmann I, Besler C (2019). Acute and Short-Term Results of Transcatheter Edge-to-Edge Repair for Severe Tricuspid Regurgitation Using the MitraClip XTR System. *JACC. Cardiovascular Interventions*.

[77] Taramasso M, Gavazzoni M, Pozzoli A, Alessandrini H, Latib A, Attinger-Toller A (2020). Outcomes of TTVI in Patients With Pacemaker or Defibrillator Leads: Data From the TriValve Registry. *JACC. Cardiovascular Interventions*.

[78] Russo G, Badano LP, Adamo M, Alessandrini H, Andreas M, Braun D (2023). Characteristics and outcomes of patients with atrial versus ventricular secondary tricuspid regurgitation undergoing tricuspid transcatheter edge-to-edge repair - Results from the TriValve registry. *European Journal of Heart Failure*.

[79] Barbieri F, Mattig I, Beyhoff N, Thevathasan T, Romero Dorta E, Skurk C (2023). Procedural success of transcatheter annuloplasty in ventricular and atrial functional tricuspid regurgitation. *Frontiers in Cardiovascular Medicine*.

[80] Galloo X, Dietz MF, Fortuni F, Prihadi EA, Cosyns B, Delgado V (2023). Prognostic implications of atrial vs. ventricular functional tricuspid regurgitation. *European Heart Journal*.

[81] Mutlak D, Aronson D, Lessick J, Reisner SA, Dabbah S, Agmon Y (2009). Functional tricuspid regurgitation in patients with pulmonary hypertension: is pulmonary artery pressure the only determinant of regurgitation severity?. *Chest*.

[82] Neuhold S, Huelsmann M, Pernicka E, Graf A, Bonderman D, Adlbrecht C (2013). Impact of tricuspid regurgitation on survival in patients with chronic heart failure: unexpected findings of a long-term observational study. *European Heart Journal*.

[83] Arnold SV, Chinnakondepalli KM, Spertus JA, Magnuson EA, Baron SJ, Kar S (2019). Health Status After Transcatheter Mitral-Valve Repair in Heart Failure and Secondary Mitral Regurgitation: COAPT Trial. *Journal of the American College of Cardiology*.

[84] Lee JW, Song JM, Park JP, Lee JW, Kang DH, Song JK (2010). Long-term prognosis of isolated significant tricuspid regurgitation. *Circulation Journal*.

[85] Asmarats L, Puri R, Latib A, Navia JL, Rodés-Cabau J (2018). Transcatheter Tricuspid Valve Interventions: Landscape, Challenges, and Future Directions. *Journal of the American College of Cardiology*.

[86] Casaclang-Verzosa G, Gersh BJ, Tsang TSM (2008). Structural and functional remodeling of the left atrium: clinical and therapeutic implications for atrial fibrillation. *Journal of the American College of Cardiology*.

